# They Might Be Giants: Does Syncytium Formation Sink or Spread HIV Infection?

**DOI:** 10.1371/journal.ppat.1006099

**Published:** 2017-02-02

**Authors:** Alex A. Compton, Olivier Schwartz

**Affiliations:** 1 Virus & Immunity Unit, Institut Pasteur, Paris, France; 2 CNRS-URA 3015, Paris, France; 3 Vaccine Research Institute, Creteil, France; University of Kentucky, UNITED STATES

## Introduction

While less appreciated than the ubiquitous process of cell fission (division), cell fusion events play crucial roles in all walks of life. In vertebrates, the multinucleated product of cell—cell fusion, referred to as a syncytium, is central to the structure and function of tissue types like skeletal muscle fibers and the fetal—maternal barrier in the placenta [1]. Syncytia have also been linked with cellular pathology in states of disease, such as HIV/AIDS. In this Pearl, we review and reconsider the controversial roles that cellular syncytia play in the replication and pathogenesis of HIV-1 infection. Furthermore, we describe research highlighting viral and cellular regulators of this cell—cell fusion activity. Overall, our aim is to provide a scientific framework necessary for the further study and appreciation of what happens when viruses bring cells together (literally).

## Fiction or Fact: Early In Vitro and In Vivo Observations

A major functional component of HIV and related retroviruses is the Envelope glycoprotein (Env), which is embedded in the viral lipid bilayer membrane and is used for attachment to surface receptors on host cells. Env also encodes the machinery necessary to carry out the virus—cell fusion sequence. In addition to its role in initiating infection, Env protein synthesized de novo in the infected cell is trafficked to the plasma membrane to allow incorporation into assembling virus particles. As a result, infected cells are decorated with non-negligible amounts of viral Env protein. Early reports characterizing the behavior of the AIDS virus in tissue culture systems demonstrated that cell surface-associated Env can engage receptors on neighboring cells and trigger cell—cell fusion events, giving rise to giant, multinucleated cells [2, 3]. Thus, in addition to driving fusion between viral and cellular membranes, viral Env can trigger host membranes to directly fuse with one another. The capacity to form syncytia in T cell lines was adopted as an early classification system (syncytia-inducing [SI] versus non-syncytia-inducing [NSI]) to distinguish different HIV-1 strains. Since HIV-induced syncytia can harbor large amounts of virus yet undergo lysis soon after their formation, these cell masses were considered to play a part in the characteristic loss of CD4+ helper T cells in HIV/AIDS disease progression [4, 5]. Reports of syncytium detection in central nervous tissue of individuals with AIDS-related encephalopathy lent credence to their possible involvement in virus pathogenesis [6, 7], as did other studies in lymphoid tissues [8, 9]. However, the cells at the origin of these in vivo manifestations of syncytia were found to be macrophages or dendritic cells, while evidence for similar events occurring in T cells was lacking. As a result, many remained unconvinced that T cell syncytia represented something other than in vitro curiosities, referring to them as “fusion sinks” that do not promote virus replication or spread. It would take decades before a fresh look would shed new light on the functional significance of cell—cell fusion in HIV infection.

## Downsizing the Functional T Cell Syncytium Using Humanized Mice

**Fig 1:**
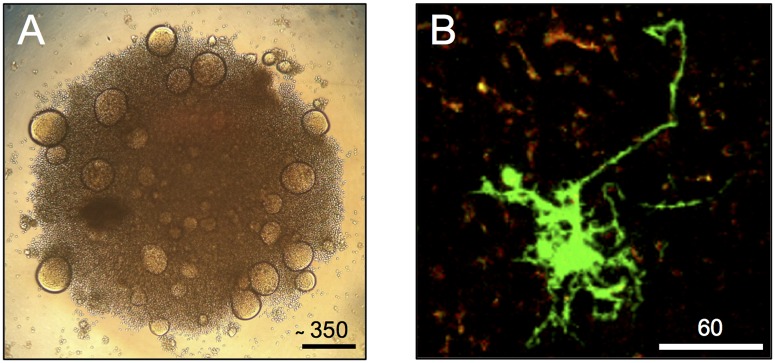
Out with the old, in with the new. (A) SupT1 T cells were incubated with HIV-1 in a 96-well plate. At two days post-infection, cells were visualized with a light microscope at 10x magnification. Scale bars are in μm. Photo taken with the iPhone 4. (B) BLT humanized NOD-SCID mice were infected with HIV-1-GFP via footpad injection. Multiphoton intravital microscopy of the draining popliteal lymph node was performed at six days post-infection. This micrograph of HIV-infected cells (green) is a maximum intensity projection of 11 z-stacks, spaced 4 μm apart. Red-orange signal corresponds to autofluorescent tissue structures. This image is a generous gift from Thomas Murooka and Thorsten Mempel.

At the turn of the century, the first direct evidence emerged that HIV-positive lymphocytes can fuse in vivo. In histological studies of lymphoid tissues rich in densely packed lymphocytes, multinucleated T cells showing signs of productive HIV-1 infection were observed [10]. Importantly, the multinucleated cells identified in these studies were much smaller (containing no more than five nuclei) compared to those described in vitro (containing up to hundreds of nuclei) (Fig 1A), suggesting that syncytia formation follows a different set of rules in living tissues. Nonetheless, the functional significance of these entities was unclear and would remain so until new technologies were adapted to the problem. With “humanized” mice harboring human lymphoid tissues came the development of a tractable in vivo model system for HIV-1 pathogenesis. A seminal article combined intravital imaging with viruses encoding fluorescent tags to enable the tracking of virus—cell interactions in real time. In contrast to the immobile, spherical giants observed in vitro, the syncytia recorded in living mice took on a slimmer size and a serpentine shape [11]. The authors observed that, in lymph nodes of infected mice, highly elongated, infected T cells connected by long membrane tethers formed in a manner that depended on HIV-1 Env expression, suggestive of Env-mediated cell—cell fusion. It was concluded that, following peripheral injection of the virus, infected T cells migrate to remote lymphoid sites and likely promote virus spread.

Further work documented and quantified small, elongated syncytia in an HIV-1 infected humanized mouse model and provided important functional characterization in an in vitro system in which these structures could be reproduced [12]. Here, small syncytia were observed using a 3-D hydrogel cell culture system consisting solely of T cells, suggesting that lymphocytes are sufficient to form such cell—cell fusions in vivo. More live cell tracking revealed that small syncytia are highly motile and can transfer the virus to uninfected lymphocytes through transient contacts without undergoing further fusion.

Together, these publications suggest that small, virus-producing syncytia may be more common in the lymph nodes of infected humans than previously thought. Of all the HIV-1 infected T cells observed in situ, approximately 20% were multinucleated, and most contained only two nuclei. Interestingly, larger syncytia were observed at later time points following infection [13]. Thus, HIV-1 induces cell—cell fusion to produce multinucleated cells of limited size, which may progressively increase in mass over time (Fig 1B). The presence of thin membrane extensions between nuclei may explain why these relatively small syncytia are not readily detectable in fixed tissue samples. Moreover, their extensive half-life and their ability to interact with uninfected T cells suggest that they likely contribute to virus dissemination in vivo. Similar observations were drawn in another study, which included an additional finding that HIV-1-infected T cells, including syncytia, behave in ways so as to promote multicopy virus transfer between cells in vivo [14].

Studies of virus infection in living cells and tissues unveil a number of important insights regarding the behavior of infected cells, but they are equally informative for what they do not reveal. T cell syncytia can form and persist in the setting of HIV-1 infection, yet they remain a minority among the totality of infected T cells. Moreover, the transitory contacts that form between syncytia and uninfected lymphocytes indicate that Env-mediated fusion is subject to temporospatial regulation.

## Intrinsic Regulation of Virus-Mediated Cell—Cell Fusion Events

**Fig 2:**
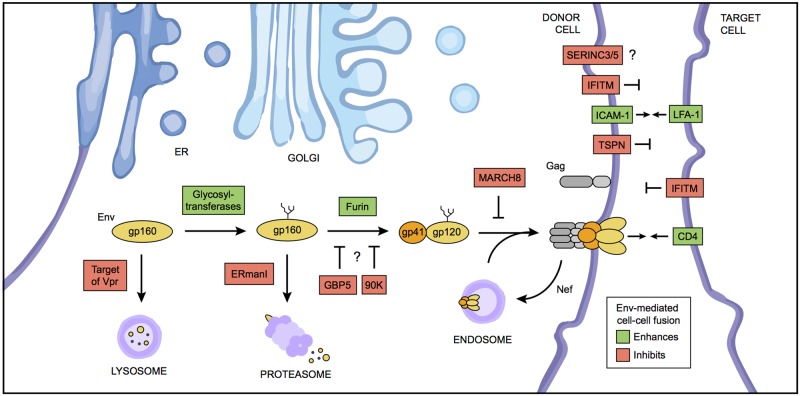
Host- and virus-mediated regulation of cell—cell fusion during HIV infection. A simplified schematic is shown of the events shaping HIV Env glycoprotein biosynthesis in the ER and Golgi and the numerous cellular factors encountered along the way. Factors colored in green contribute positively to Env expression, maturation, localization, and/or direct fusogenic capacity, while factors colored in red are inhibitory to one or more of these processes. Arrows and blunt-end lines indicate proteins that promote or inhibit fusion, respectively, between the infected (donor) cell and a neighboring target cell. Question marks are placed where a mechanistic understanding is lacking: GBP5 and 90K expression both lead to the accumulation of gp160 Env, but direct inhibition of gp120–gp41 formation has yet to be demonstrated. Similarly, it is unknown whether SERINC3/5 inhibits Env-mediated cell—cell fusion. TSPN, tetraspanins. IFITM, interferon-induced transmembrane proteins.

It has long been recognized that, in tightly packed environments, HIV-infected T cells engage neighboring cells and form a “virological synapse,” named as such because it resembles the immunological synapse that T cells form with antigen-presenting cells [15] (reviewed in another Pearl [16]). Characterized by a coordinated mobilization of host and viral proteins, virological synapse formation results in efficient cell-to-cell transfer of virions in the absence of cell fusion. In fact, while certain cellular proteins like LFA-1 and ICAM proteins facilitate cell—cell adhesion during this process, other components of the synapse appear to actively suppress the fusion of apposing cell membranes (Fig 2) [16]. Cellular proteins enriched at the virological synapse, such as tetraspanins CD9 and CD63 (as well as the actin organizer, ezrin), have been shown to inhibit syncytia formation [17–19]. Furthermore, Env protein may be actively down-regulated from the surface of the infected cell [20]. Together, this evidence has been used to suggest that virus-mediated cell—cell fusion (and, by extension, syncytia) may not facilitate virus dissemination.

While certain processes controlling glycoprotein fusion of other viruses do not pertain to HIV (such as pH-dependent triggering), another mechanism of fusion regulation is hard-wired into HIV-1 virions. In immature particles, the structural Gag precursor protein inhibits Env-mediated fusion by interacting with the cytoplasmic tail of the gp41 subunit. Once Gag is cleaved during virion budding and maturation, Env fusion activity is restored [21–23]. Moreover, deletion of the gp41 cytoplasmic tail results in rampant syncytia formation between infected cells in culture. Overall, a large amount of Env at the infected cell surface or in budding virions is not fully competent for fusion [24]. When considering the recent in vivo observations of infected, multinucleated T cells that may assist in virus dissemination, it is possible that limited syncytia formation among a minority of infected T cells can encourage virus spread without compromising transmission via the virological synapse. It is also possible that the balance between the formation and suppression of syncytia is weighted differently depending on (1) the phase of HIV-1 infection; (2) the tissue site of replication; and (3) the induction of antiviral innate immunity. The viral fusion step itself may represent a “danger” signal capable of triggering a type-I interferon or inflammatory response, the effects of which may be sensed by neighboring cells [25, 26]. In that regard, limiting the extent of syncytia formation may allow the virus to limit the activation of host responses.

## Tipping the Balance Towards Protection

While processes governing virus-mediated cell—cell fusion are affected by extracellular factors, such as Env-specific antibodies, they are also subject to a cellular landscape that changes once virus infection is detected or “sensed.” Every cell is equipped with a cell-autonomous innate immune system consisting of nucleic acid sensors and antiviral effector proteins that are mobilized in response to invading viruses [27]. Several host factors have emerged recently as relevant players in virus-mediated fusion events, with each directly targeting Env biosynthesis or inhibiting Env-dependent fusion.

The interferon-induced transmembrane (IFITM) proteins occupy cellular membranes and inhibit virus—cell fusion by altering the properties of lipid bilayers in which they reside and also inhibit the fusogenicity of HIV-1 particles [28, 29]. Accordingly, IFITM proteins inhibit syncytia formation in vitro [28, 30] (Fig 2). Another pair of transmembrane proteins, the serine incorporators SERINC3 and SERINC5, also inhibits HIV-1 virion fusogenicity [31, 32]. It is not yet clear whether SERINC3 and SERINC5 exhibit an anti-cell-fusion activity like IFITM proteins and tetraspanins. Virus-mediated fusion is under further threat by other cellular factors that degrade or down-modulate Env at different steps in its trafficking pathway: the E3 ubiquitin ligase MARCH8, the ER-associated mannosidase ERManI, the guanosine triphosphatase GBP5, an unknown interferon-induced host factor in macrophages, and a galactin-3 binding protein termed 90K [33–35] (Fig 2). Working out the precise mechanisms by which these many antiviral factors act will enable future efforts aimed at enhancing the restrictive potential of cells in vivo.

## Conclusions and Outstanding Questions

By combining intravital imaging of mice with advancements in gene modification and immunotherapy, the identification of host factors that control the formation or behavior of multinucleated cells will be possible. Furthermore, the perturbation or enhancement of small, mobile syncytia will define their ultimate importance to HIV dissemination. Adapting the same living systems for use with other virus strains with different origins and host tropisms (such as simian immunodeficiency virus [SIV]) may also reveal the extent to which the induction of cell—cell fusion is conserved during lentivirus evolution. Likewise, the in vivo study of other viruses known to induce syncytia in vitro, such as nonlentiviral retroviruses (T lymphotropic virus and foamy virus) [36] and RNA viruses (measles virus and respiratory syncytial virus) [26, 37], will determine how these striking cellular structures are relevant to the spread, sensing, and pathology of other infections.

## References

[1] Chen EH, Grote E, Mohler W, Vignery A. Cell-cell fusion. FEBS Lett. 2007;581(11):2181–93. 10.1016/j.febslet.2007.03.033 17395182

[2] Lifson JD, Reyes GR, McGrath MS, Stein BS, Engleman EG. AIDS retrovirus induced cytopathology: giant cell formation and involvement of CD4 antigen. Science. 1986;232(4754):1123–7. 3010463 10.1126/science.3010463

[3] Kowalski M, Potz J, Basiripour L, Dorfman T, Goh WC, Terwilliger E, et al Functional regions of the envelope glycoprotein of human immunodeficiency virus type 1. Science. 1987;237(4820):1351–5. 3629244 10.1126/science.3629244

[4] Sylwester A, Murphy S, Shutt D, Soll DR. HIV-induced T cell syncytia are self-perpetuating and the primary cause of T cell death in culture. J Immunol. 1997;158(8):3996–4007. 9103471

[5] Sylwester A, Daniels K, Soll DR. The invasive and destructive behavior of HIV-induced T cell syncytia on collagen and endothelium. J Leukoc Biol. 1998;63(2):233–44. 9468282 10.1002/jlb.63.2.233

[6] Koenig S, Gendelman HE, Orenstein JM, Canto MCD, Pezeshkpour GH, Yungbluth M, et al Detection of AIDS virus in macrophages in brain tissue from AIDS patients with encephalopathy. Science. 1986;233(4768):1089–93. 3016903 10.1126/science.3016903

[7] Pumarola Sune T, Navia BA, Price RW, Cordon Cardo C, Cho ES. HIV antigen in the brains of patients with the AIDS dementia complex. Ann Neurol. 1987;21(5):490–6. 10.1002/ana.410210513 3296948

[8] Rinfret A, Latendresse H, Lefebvre R, St-Louis G, Jolicoeur P, Lamarre L. Human immunodeficiency virus-infected multinucleated histiocytes in oropharyngeal lymphoid tissues from two asymptomatic patients. Am J Pathol. 1991;138(2):421–6. 1992767 PMC1886194

[9] Frankel SS, Wenig BM, Burke AP, Mannan P, Thompson LD, Abbondanzo SL, et al Replication of HIV-1 in dendritic cell-derived syncytia at the mucosal surface of the adenoid. Science. 1996;272(5258):115–7. 8600520 10.1126/science.272.5258.115

[10] Orenstein JM. In vivo cytolysis and fusion of human immunodeficiency virus type 1-infected lymphocytes in lymphoid tissue. J Infect Dis. 2000;182(1):338–42. 10.1086/315640 10882620

[11] Murooka TT, Deruaz M, Marangoni F, Vrbanac VD, Seung E, von Andrian UH, et al HIV-infected T cells are migratory vehicles for viral dissemination. Nature. 2013;490(7419):283–7. 10.1038/nature11398 PMC3470742 22854780

[12] Symeonides M, Murooka T, Bellfy L, Roy N, Mempel T, Thali M. HIV-1-Induced Small T Cell Syncytia Can Transfer Virus Particles to Target Cells through Transient Contacts. Viruses. 2015;7(12):6590–603. 10.3390/v7122959 26703714 PMC4690882

[13] Murooka TT, Sharaf RR, Mempel TR. Large Syncytia in Lymph Nodes Induced by CCR5-Tropic HIV-1. AIDS Res Hum Retroviruses. 2015;31(5):471–2. 10.1089/aid.2014.0378 25835064 PMC4426294

[14] Law KM, Komarova NL, Yewdall AW, Lee RK, Herrera OL, Wodarz D, et al In Vivo HIV-1 Cell-to-Cell Transmission Promotes Multicopy Micro-compartmentalized Infection. Cell Rep. 2016;15(12):2771–83. 10.1016/j.celrep.2016.05.059 27292632

[15] Igakura T, Stinchcombe JC, Goon PKC, Taylor GP, Weber JN, Griffiths GM, et al Spread of HTLV-I between lymphocytes by virus-induced polarization of the cytoskeleton. Science. 2003;299(5613):1713–6. 10.1126/science.1080115 12589003

[16] Alvarez RA, Barría MI, Chen BK. Unique Features of HIV-1 Spread through T Cell Virological Synapses. PLoS Pathog. 2014;10(12):e1004513–4. 10.1371/journal.ppat.1004513 25522148 PMC4270788

[17] Symeonides M, Lambelé M, Roy N, Thali M. Evidence Showing that Tetraspanins Inhibit HIV-1-Induced Cell-Cell Fusion at a Post-Hemifusion Stage. Viruses. 2014;6(3):1078–90. 10.3390/v6031078 24608085 PMC3970140

[18] Roy NH, Lambele M, Chan J, Symeonides M, Thali M. Ezrin is a Component of the HIV-1 Virological Presynapse and Contributes to the Inhibition of Cell-Cell Fusion. J Virol. 2014: JVI.00550-14. 10.1128/JVI.00550-14 PMC4054451 24760896

[19] Weng J, Krementsov DN, Khurana S, Roy NH, Thali M. Formation of syncytia is repressed by tetraspanins in human immunodeficiency virus type 1-producing cells. J Virol. 2009;83(15):7467–74. 10.1128/JVI.00163-09 19458002 PMC2708618

[20] Schwartz O, Rivière Y, Heard JM, Danos O. Reduced cell surface expression of processed human immunodeficiency virus type 1 envelope glycoprotein in the presence of Nef. J Virol. 1993;67(6):3274–80. 8497051 10.1128/jvi.67.6.3274-3280.1993 PMC237668

[21] Murakami T, Ablan S, Freed EO, Tanaka Y. Regulation of human immunodeficiency virus type 1 Env-mediated membrane fusion by viral protease activity. J Virol. 2004;78(2):1026–31. 10.1128/JVI.78.2.1026-1031.2004 14694135 PMC368813

[22] Roy NH, Chan J, Lambele M, Thali M. Clustering and Mobility of HIV-1 Env at Viral Assembly Sites Predict Its Propensity To Induce Cell-Cell Fusion. J Virol. 2013;87(13):7516–25. 10.1128/JVI.00790-13 23637402 PMC3700267

[23] Wyma DJ, Jiang J, Shi J, Zhou J, Lineberger JE, Miller MD, et al Coupling of Human Immunodeficiency Virus Type 1 Fusion to Virion Maturation: a Novel Role of the gp41 Cytoplasmic Tail. J Virol. 2004;78(7):3429–35. 10.1128/JVI.78.7.3429-3435.2004 15016865 PMC371074

[24] Chojnacki J, Staudt T, Glass B, Bingen P, Engelhardt J, Anders M, et al Maturation-Dependent HIV-1 Surface Protein Redistribution Revealed by Fluorescence Nanoscopy. Science. 2012;338(6106):524–8. 10.1126/science.1226359 23112332

[25] Holm CK, Jensen SorB, Jakobsen MR, Cheshenko N, Horan KA, Moeller HB, et al Virus-cell fusion as a trigger of innate immunity dependent on the adaptor STING. Nat Immunol. 2012;13(8):737–43. 10.1038/ni.2350 22706339 PMC3411909

[26] Herschke F, Plumet S, Duhen T, Azocar O, Druelle J, Laine D, et al Cell-cell fusion induced by measles virus amplifies the type I interferon response. J Virol. 2007;81(23):12859–71. 10.1128/JVI.00078-07 17898060 PMC2169089

[27] Simon V, Bloch N, Landau NR. Intrinsic host restrictions to HIV-1 and mechanisms of viral escape. Nat Immunol. 2015;16(6):546–53. 10.1038/ni.3156 25988886 PMC6908429

[28] Compton AA, Bruel T, Porrot F, Mallet A, Sachse M, Euvrard M, et al IFITM Proteins Incorporated into HIV-1 Virions Impair Viral Fusion and Spread. Cell Host Microbe. 2014;16(6):736–47. 10.1016/j.chom.2014.11.001 25464829 PMC7104936

[29] Tartour K, Appourchaux R, Gaillard J, Nguyen X-N, Durand S, Turpin J, et al IFITM proteins are incorporated onto HIV-1 virion particles and negatively imprint their infectivity. Retrovirology. 2014;11(1):103. 25422070 10.1186/s12977-014-0103-y PMC4251951

[30] Bailey CC, Zhong G, Huang IC, Farzan M. IFITM-Family Proteins: The Cell's First Line of Antiviral Defense. Annu Rev Virol. 2014;1(1):261–83. 25599080 10.1146/annurev-virology-031413-085537 PMC4295558

[31] Rosa A, Chande A, Ziglio S, De Sanctis V, Bertorelli R, Goh SL, et al HIV-1 Nef promotes infection by excluding SERINC5 from virion incorporation. Nature. 2015;526(7572):212–7. 10.1038/nature15399 26416734 PMC4861059

[32] Usami Y, Wu Y, Göttlinger HG. SERINC3 and SERINC5 restrict HIV-1 infectivity and are counteracted by Nef. Nature. 2015;526(7572):218–23. 10.1038/nature15400 26416733 PMC4600458

[33] Krapp C, Hotter D, Gawanbacht A, McLaren PJ, Kluge SF, Stürzel CM, et al Guanylate Binding Protein (GBP) 5 Is an Interferon- Inducible Inhibitor of HIV-1 Infectivity. Cell Host Microbe. 2016;19(4):504–14. 10.1016/j.chom.2016.02.019 26996307

[34] Wei W, Yu XF. HIV-1 Envelope Under Attack. Trends Microbiol. 2016;24(3):164–6. 10.1016/j.tim.2016.01.004 26803378

[35] Goffinet C. Cellular Antiviral Factors that Target Particle Infectivity of HIV-1. Curr HIV Res. 2016;14(3):211–6. 26674651 10.2174/1570162X14666151216145521 PMC5403965

[36] Coffin JM. Retroviruses: CSHL Press; 1997 843 p.

[37] Tian J, Huang K, Krishnan S, Svabek C, Rowe DC, Brewah Y, et al RAGE inhibits human respiratory syncytial virus syncytium formation by interfering with F-protein function. J Gen Virol. 2013;94(Pt 8):1691–700. 10.1099/vir.0.049254-0 23559480 PMC3749528

